# Comprehensive Qualitative Ingredient Profiling of Chinese Herbal Formula Wu-Zhu-Yu Decoction via a Mass Defect and Fragment Filtering Approach Using High Resolution Mass Spectrometry

**DOI:** 10.3390/molecules21050664

**Published:** 2016-05-19

**Authors:** Huarong Xu, Huibin Niu, Bosai He, Chang Cui, Qing Li, Kaishun Bi

**Affiliations:** 1 National and Local Joint Engineering Laboratory for Key Technology of Chinese Material Medica Quality Control, School of Pharmacy, Shenyang Pharmaceutical University, 103 Wenhua Road, Shenyang 110016, China; huarongxu@outlook.com (H.X.); 15009889276@163.com (H.N.); jesse83@163.com (B.H.); lqyxm@hotmail.com (Q.L.); 2 Liaoning Institute of Analytical Science, 103 Wanliutang Rd., Shenyang 110015, China; changcuiln@yeah.net

**Keywords:** ingredients qualitative analysis, Wu-Zhu-Yu decoction, Traditional Chinese Medicine formula, high resolution mass spectrometry

## Abstract

The Wu-Zhu-Yu decoction is a traditional Chinese medicine formula for the treatment of headache. To reveal its material basis, a rapid and reliable liquid chromatography-high resolution mass spectrometry method was established for comprehensive profiling of the chemical ingredients in the Wu-Zhu-Yu decoction. The method was used on a quadrupole time-of-flight mass spectrometer along with an advanced data processing procedure consisting of mass accuracy screening, mass defect filtering and fragment filtering. After eliminating interference with a filtering approach, the MS data profiling was made more distinct and accurate. With the optimized conditions of only 35 min LC separation and single sample injection of each positive or negative ion mode, a total of 168 compounds were characterized, including 23 evodiamine and its analogous alkaloids, 12 limonoids, 17 gingerols, 38 ginsenosides, 15 flavonoids, 16 organic acids, 14 alkaloids, 5 saponins, 3 2,2-dimethylchromenes and 25 other compounds. The fragmentation patterns of representative compounds were illustrated as well. Integrative qualitative analysis of the Wu-Zhu-Yu decoction by high resolution mass spectrometry was accomplished and reported for the first time. The study demonstrated that the established method was a powerful and reliable strategy for comprehensive detection and would be widely applicable for identification of complicated components from herbal prescriptions, and may provide a basis for chemical analysis of other complex mixtures.

## 1. Introduction

Globalization and modernization have become principle targets of traditional Chinese medicine (TCM), in particular herbal drugs and prescriptions. Herbal prescriptions have been widely applied and their definitive therapeutic effects are based on synergistic contribution of multiple ingredients which can lead to a multi target treatment [1,2,3,4]. Therefore, with the expanded usage of TCM worldwide, investigation with scientific, effective and reliable methodologies has become a priority task to ensure identity, quality and consistency. In the past decades, natural products have been one of the main resources for screening lead compounds in drug discovery [5,6]. Consequently, in order to reveal the pharmacological mechanism of TCM, accurate detection and identification of active ingredients are the preliminary goal.

The Wu-Zhu-Yu decoction (WZYD) is a classic TCM formula which has been extensively used for the treatment of headache, gastrointestinal disorders, *etc.* in both traditional and modern clinical applications in China [7,8]. The formula contains four traditional Chinese *materia medica*, Euodiae Fructus (dried and nearly ripe fruit of *Euodia rutaecarpa* (Juss.) Benth.), Zingiberis Rhizoma Recens (fresh rhizome of *Zingiber officinale* (Willd.) Rosc.), Ginseng Radix et Rhizoma (dried root or rhizome of *Panax ginseng* C. A. Mey.) and Jujubae Fructus (dried ripe fruit of *Ziziphus jujube* Mill.), which make the formula very difficult to recognize due to its complex components. Moreover, few studies have been reported pertaining to the comprehensive ingredient qualitative analysis of WZYD. The great structural diversity of chemical compounds made it difficult to provide good responses to all chemical components in MS analysis. In order to identify as many ingredients as possible from WZYD in a short time, it was necessary to set up a reliable and effective analytical method.

Quadrupole time-of flight (QTOF) hybrid mass spectrometry system delivers high resolution, accurate mass of both precursor and product ions for advanced qualitative workflows. In addition, comparing with other high resolution MS like Orbitrap and FT-ICR, a much higher acquisition rate up to 100 spectra/s is the most remarkable characteristics of a QTOF mass spectrometer and may lead the exploration of complex samples to greater depth [9,10,11]. These characteristics of comprehensive qualitative exploration and rapid profiling of target and unknown compounds make the QTOF MS system extraordinarily suitable for qualitative analysis of a complex herbal extract [12,13,14]. In previous reports, detection of the TCM components from full scan mass chromatograms using liquid chromatography-quadrupole time-of-flight mass spectrometry (LC-QTOF MS) used to be performed manually with low efficiency. Normally, relatively low signals from a complicated background or matrix were difficult and fallible in the identification process. Abundant information was usually generated by tens of thousands of MS ions and thousands of chromatographic peaks from any compounds present in the sample as well as sample matrix itself in the detection of herbal extracts [15,16]. Consequently, a powerful data-processing strategy using the in-built mass defect filtering (MDF) and fragment filtering function in Sciex PeakView^®^ software (version 2.2, Sciex, Redwood City, CA, USA) was adopted to analyze the MS data. Overall, an efficient and generally applicable approach for qualitative analysis of WZYD based on LC separation followed by high resolution and accurate mass MS and MS/MS detection was successfully established. Meanwhile, a rapid and reliable data profiling procedure with MDF and fragment filtering was highlighted. It is expected that the method and result of the study would provide a helpful methodological and chemical basis for further research on WZYD and other herbal extracts.

## 2. Results and Discussion

### 2.1. Optimization of LC Q-TOF MS Conditions

In order to obtain chromatograms with better separation and higher MS performance, different mobile phase systems including different organic phase and different types and amounts of eluent additives were tested to optimize the LC-MS behavior. As a result, the optimal solvent systems consisting of 0.1% formic acid in acetonitrile and 0.1% formic acid aqueous solution on the optimized gradient showed excellent separation and abundant signals in both positive and negative ion modes. The MS conditions, ion source temperature, ion spray voltage, nebulizer gas, heater gas, curtain gas, and collision energy, were optimized in order to achieve higher resolution and sensitivity to all ingredients in WZYD. An information-dependent acquisition (IDA) algorithm was used to explore complex samples in greater depth. 8 precursors per second were selected to generate MS/MS data. High scan rate IDA mode allowed high-resolution and accurate mass TOF MS and MS/MS information to be collected simultaneously in a single run. Consequently, outstanding efficiency of ionization and satisfactory sensitivity were achieved through the adopted LC-MS conditions.

### 2.2. Peaks Detection and Confirmation of WZYD by LC Q-TOF MS

**Figure 1:**
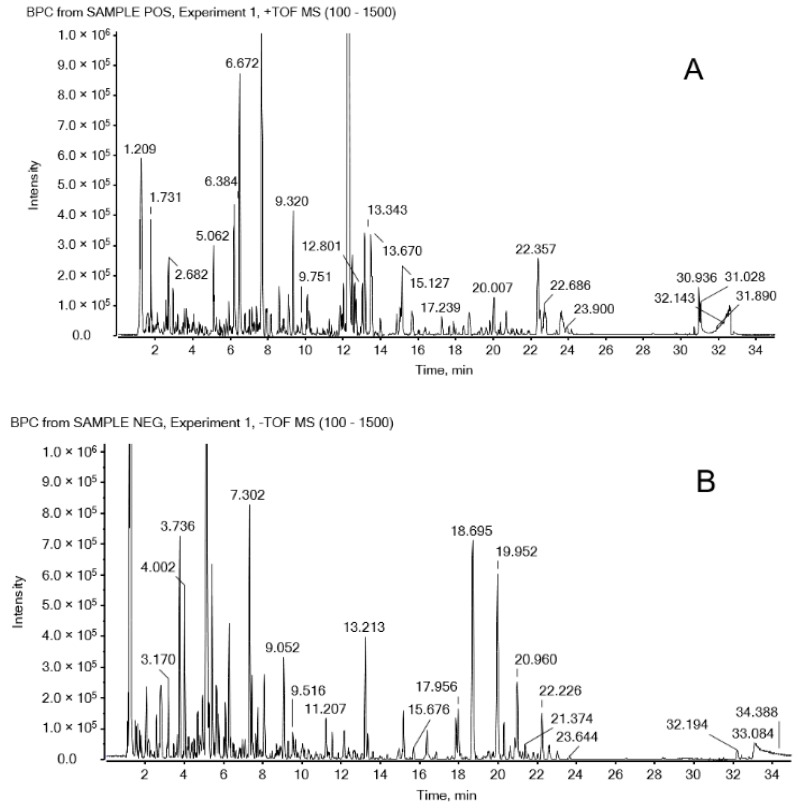

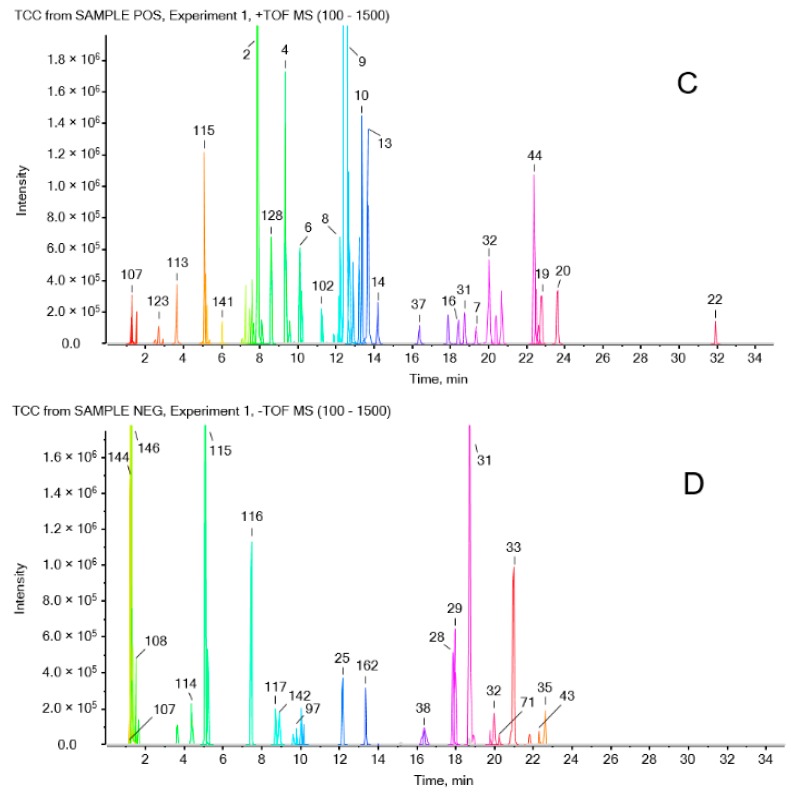
BPC of Wu-Zhu-Yu decoction in (**A**) positive and (**B**) negative ion modes by LC-QTOF MS, and TCC in (**C**) positive and (**D**) negative ion modes. The labels of the TCC peaks were all corresponding to the compound numbers in Table S1.

After the optimization, a simple LC-Q-TOF MS method was developed to detect ingredients in WZYD. The base peak chromatograms (BPC) of positive and negative ion modes in Figure 1 show that most of the chromatographic peaks were well separated under the optimized gradient elution conditions. However, matrix-derived signals made finding characteristic compounds quite challenging. Only a few peaks could be distinguished from the background in TIC (total ion chromatogram) and BPC, while many signals were submerged. Therefore, the MDF and fragment filtering approach were used to simplify the identification.

In the full scan mass spectra, most of the authentic compounds exhibited [M + H]^+^ or [M + Na]^+^ ions in positive ion mode and [M − H]^−^ or [M + HCOOH-H]^−^ in negative ion mode. All the compounds were preliminarily characterized based on their mass accuracy of precursor ions (<5 ppm), isotopic pattern, fragmentation profile, and retention behavior referring to previous reports using PeakView^®^ (version 2.2) and MasterView™ (version 1.1) software (Sciex, Redwood City, CA, USA). Totally, 168 compounds were characterized, including 23 evodiamine and its analogous alkaloids [17,18,19,20], 12 limonoids [21,22,23], 17 gingerols, 38 ginsenosides [24,25,26,27], 15 flavonoids, 16 organic acids, 14 alkaloids, 5 saponins, 3 2,2-dimethylchromenes and 25 other compounds. Information on precursor and product ion, error of mass accuracy and retention time (t_R_) of the 168 compounds is shown in Table S1, and the total compound chromatograms (TCC) generated by the extracted ion chromatograms (XIC) of the 168 compounds are shown in Figure 1.

#### 2.2.1. Identification of Evodiamine and Its Analogous Alkaloids in WZYD

**Figure 2:**
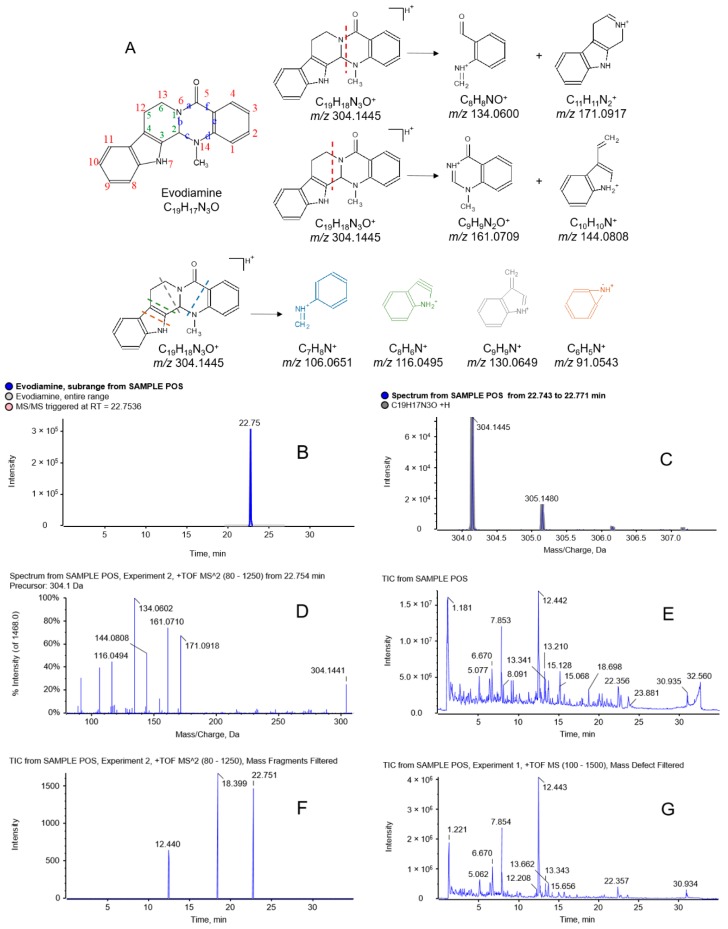
Fragmentation pattern of evodiamine and its analogous alkaloids. (**A**) Structure and fragmentation pathways of evodiamine, (**B**) XIC of evodiamine, (**C**) precursor ion TOF MS spectrum of evodiamine, (**D**) product ion MS/MS spectrum of evodiamine, (**E**) TIC of TOF MS scan of WZYD in positive ion mode, (**F**) TIC of CID experiment 2 after fragment filtering, and (**G**) TIC of TOF MS scan after MDF.

Evodiamine and its analogous alkaloids are the most significant compounds with multiple bioactivities in Euodiae Fructus, the principle herb of the formula. Evodiamine, one of the most abundant and widely reported alkaloids in the herb, was selected to characterize the fragmentation pathways of the series compounds. The XIC, precursor and product ion spectrums are shown in Figure 2. Alkaloids normally show excellent [M + H]^+^ signals in positive ion mode. [M + H]^+^ of *m/z* 304.1445 was detected as the precursor ion of evodiamine. As shown in Figure 2A, the a bond and c bond in the quinazoline ring of the compound were cleaved into two parts after collision-induced dissociation (CID), and formed product ions of C_8_H_8_NO^+^ with *m/z* 134.0602 and C_11_H_11_N_2_^+^ with *m/z* 171.0918. In another fragmentation pattern, the cleavage happened in the N-C6 bond and C2-C3 bond of the pyridine ring, and formed product ions of C_9_H_9_N_2_O^+^ with *m/z* 161.0710 and C_10_H_10_N^+^ with *m/z* 144.0808. The two fragmentation pathways also happened in other analogous alkaloids which provided valuable clues on identifying and reconfirming other analogues. Other fragments of C_8_H_6_N^+^ with *m/z* 116.0494, C_7_H_8_N^+^ with *m/z* 106.0651 and C_9_H_9_N^+^ with *m/z* 130.0649 were formed by the CID as well.

##### MDF Approach Profiling

The theoretical mass defect 0.1444 of evodiamine was adopted as the mass defect filtering reference for preliminary screening of evodiamine-analogous alkaloids. After setting limitations of the defect tolerance of 50 mDa and the filtering mass range of 200–400 Da, a new chromatogram after mass defect filtering, shown in Figure 2G, was generated. Apparently, the noise level of the filtered chromatogram was much lower than that of the original TOF MS TIC (Figure 2E). From the filtered chromatogram, much fewer peaks were shown, so that those peaks could be targeted and systematically analyzed, which showed a powerful ability to reduce the data processing work.

##### Fragment Filtering Approach Profiling

After identifying a few well-known alkaloids such as evodiamine, rutaecarpine, dehydroevodiamine and evodiamide, the fragment ions of theoretical *m/z* 134.0600, 161.0709, 144.0802 and 106.0648 were found to be the most common fragments among these alkaloids. Therefore, these fragments were chosen as the references in the fragment filtering. As in the IDA method described above, 8 precursor ions were selected in experiment 1 of the TOF MS scan, and then we performed CID. The accurate mass of the 4 fragments mentioned above along with a mass tolerance of 5 ppm were used as the filtering criteria to the 8 CID scan experiments. The predicted compound of hydroxygoshuyuamide was used to demonstrate how the compound was identified using the filter. When the fragment filter of the fragment *m/z* 134.0600 was employed in experiment 2, a filtered chromatogram was generated (Figure 2F). Three peaks were shown in the chromatogram, two of which were the compounds already identified preliminarily by target screening. This two identified compounds of evodiamine and dehydroevodiamine were both common alkaloids in Euodiae Fructus based on previous reports. Consequently, the peak with retention time of 18.4 min was listed as an unknown compound we were interested in. Only a rough precursor ion *m/z* of 322.2 was obtained in the CID information since it was selected by the quadrupole. Afterwards, in accordance with the retention time, an accurate mean *m/z* of 322.1548 was obtained from the TOF MS TIC. The formula of C_19_H_19_N_3_O_2_ was predicted using the formula finder function. The TOF MS and MS/MS spectrums were shown in Figure S1. [M + H_2_O + H]^+^ of evodiamine used to be considered the identity of the unknown compound. In this case, the two compounds should share similar fragments. However, the results showed otherwise. After screening the identified alkaloids, goshuyuamide was found to have fragments of *m/z* 134.0605, 106.0652 and 116.0497 similar to the unknown compound. As a result, the compound was predicted as hydroxygoshuyuamide. The fragment of *m/z* 160.0762 of hydroxygoshuyuamide was predicted as the hydroxyl addition of the fragment of *m/z* 144.0799 in goshuyuamide. This demonstrated that the substitution of hydroxyl could possibly be located on the indole ring, while an accurate site for the substitute could not be confirmed. Additionally, other similar fragment filtering approaches were applied in the identification of other ingredients in WZYD.

#### 2.2.2. Identification of Limonoids in WZYD

**Figure 3:**
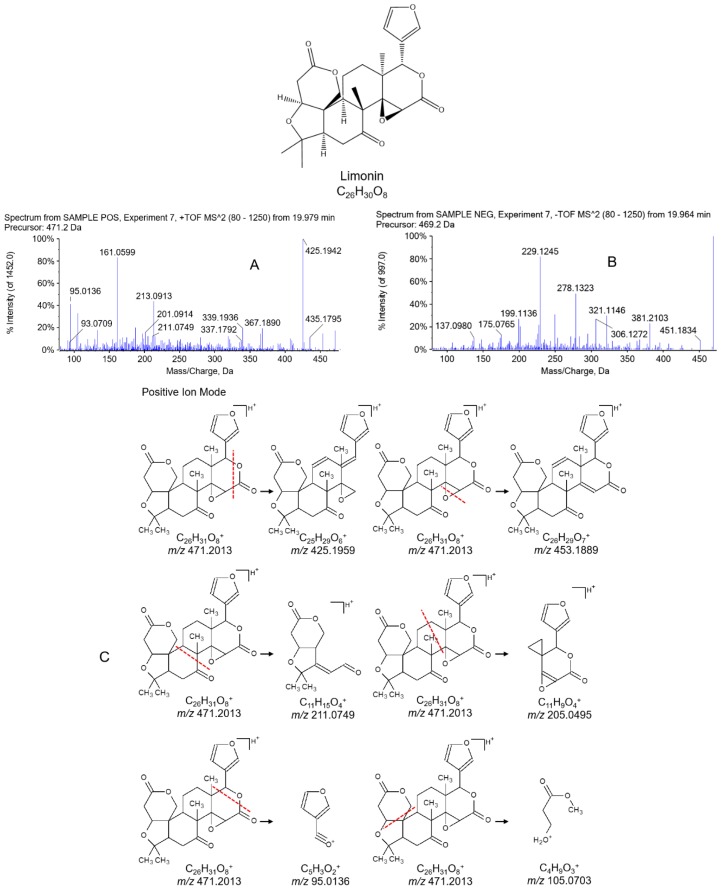

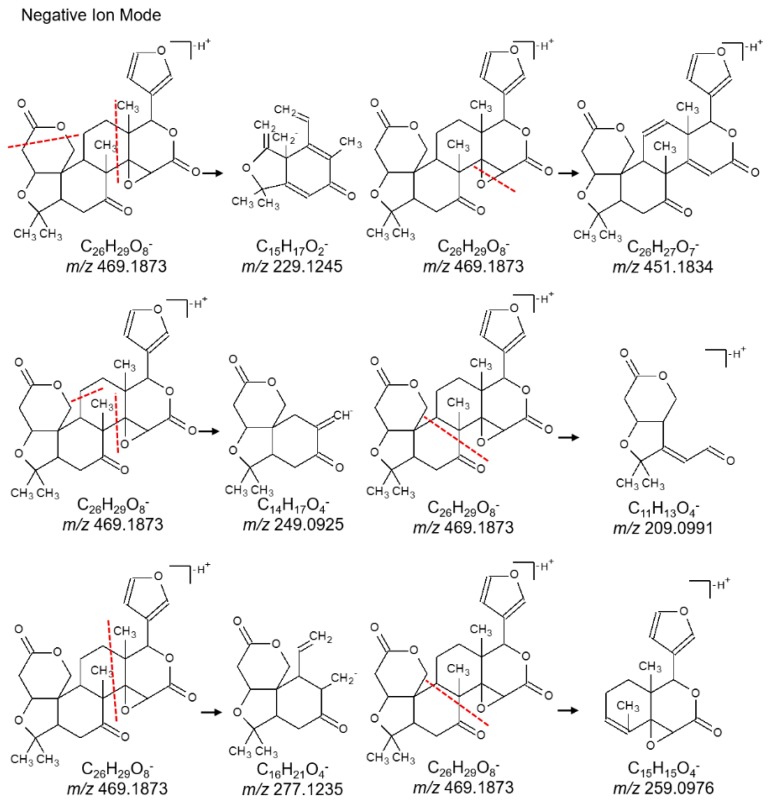
Product ion MS/MS spectrums of limonin in (**A**) positive and (**B**) negative ion modes, and (**C**) fragmentation pathways of limonin.

Limonoids, which consist of variations of the furanolactone core structure, are also key ingredients in Euodiae Fructus. Limonin, the most abundant and widely reported limonoid, was used to illustrate the fragmentation pattern (Figure 3). Quasi-molecular ions were found in both positive and negative ion modes and exhibited [M + H]^+^ of *m/z* 471.2010 as well as [M − H]^−^ of *m/z* 469.1873. In positive ion mode, the furanolactone ring in limonin would be broken to form the fragment of C_25_H_29_O_6_^+^ with *m/z* 425.1942; the furanolactone ring might also be dissociated in another pathway to generate a characteristic fragment of C_5_H_3_O_2_^+^ with *m*/*z* 95.0136. Other fragmentations shown in Figure 3A demonstrated how fragments of C_26_H_29_O_7_^+^ with *m/z* 453.1889, C_11_H_15_O_4_^+^ with *m/z* 211.0749, C_11_H_9_O_4_^+^ with *m/z* 205.0495 and C_4_H_9_O_3_^+^ with *m/z* 105.0703 were generated. In negative ion mode, the same fragments as C_26_H_29_O_7_^+^ with *m/z* 453.1889 and C_11_H_15_O_4_^+^ with *m/z* 211.0749 in positive ion mode appeared as well, producing C_26_H_27_O_7_^−^ with *m/z* 451.1834 and C_11_H_13_O_4_^−^ with *m/z* 209.0991. Other fragments of C_14_H_17_O_4_^−^ with *m/z* 249.0925, C_12_H_17_O_4_^−^ with *m/z* 225.0912, C_16_H_21_O_4_^−^ with *m/z* 277.1235 and C_15_H_15_O_4_^−^ with *m/z* 259.0976 were generated by the fragmentation pattern shown in Figure 3A. The MDF and fragment filtering approach were also applied in the limonoid identification. The mass defect 0.2013 of limonin was selected as the mass defect filtering reference along with the defect tolerance of 50 mDa and the filtering mass range of 400–600 Da. The fragments of *m/z* 95.0136 and *m/z* 105.0703 in positive ion mode were adopted as the fragment filtering references.

#### 2.2.3. Identification of Gingerols in WZYD

**Figure 4:**
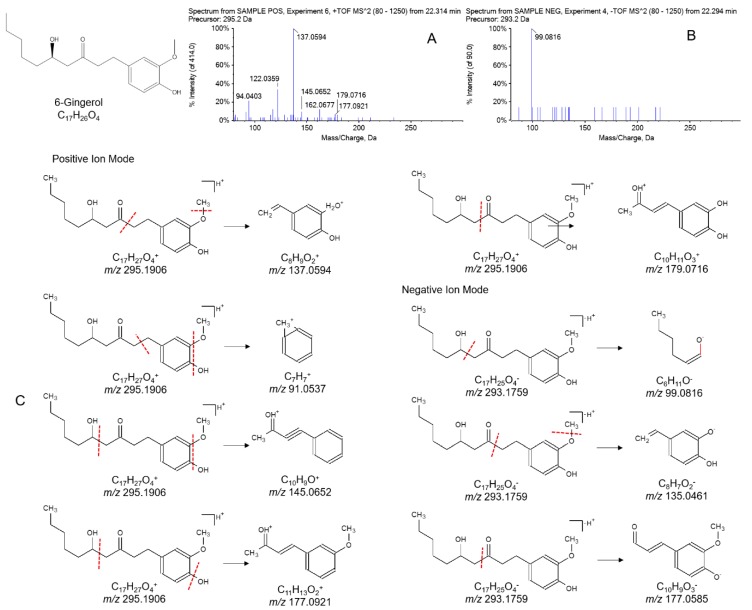
Product ion MS/MS spectrums of 6-gingerol in (**A**) positive and (**B**) negative ion modes and (**C**) fragmentation pathways of 6-gingerol.

Gingerols are active constituents which can be found in fresh ginger. Chemically, gingerols are the compounds which give chili peppers spiciness. As 6-gingerol was commonly studied, it was used to illustrate the fragmentation pattern. [M + H]^+^ of *m/z* 295.1906 and [M − H]^−^ of *m/z* 293.1759 were found in positive and negative ion modes respectively. Positive ion fragments of C_8_H_9_O_2_^+^ with *m/z* 137.0594, C_7_H_7_^+^ with *m/z* 91.0537, C_10_H_9_O^+^ with *m/z* 145.0648, C_11_H_13_O_2_^+^ with *m/z* 177.0921, and C_10_H_11_O_3_^+^ with *m/z* 179.0716 were formed in the pathways shown in Figure 4. Negative ion fragments of C_8_H_7_O_2_^−^ with *m/z* 135.0461 and C_10_H_9_O_3_^−^ with *m/z* 177.0585 have the same fragmentation pattern as their respective positive ions. The fragment of *m/z* 91.0542 was selected as the fragment filtering reference.

#### 2.2.4. Identification of Ginsenosides in WZYD

Ginsenosides are one of the most famous phytochemicals currently, and chemically, most of them are triterpenoid saponins. Compounds in this family are found almost exclusively in the plant Panax ginseng, which has a long history of use in TCM that has led to extensive studies of pharmacological effects of the compounds. Ginsenosides were not the key targets in this study since a large amount of previous studies had reported the identification and fragment patterns by mass spectrometry. Ginsenosides were found to be mainly divided into two major groups including the protopanaxadiol (PPD) type with sugar moieties attached to the C-3 with or without C-20, and the protopanaxatriol (PPT) type with sugar moieties at C-6 with or without at C-20. A pair of isomers, ginsenoside Rd and Re, were used to demonstrate the fragmentations (Figure S2). Typically, ginsenosides form [M + Na]^+^ in positive ion mode, while [M − H]^−^ and [M + HOOH − H]^−^ form in negative ion mode, but normally have a stronger signal in negative ion mode, especially in CID performance. Ginsenoside Rd, which belongs to PPD type ginsenosides, performed neutral loss of 1, 2 and 3 glucose residuals of 162.0528, one at a time, to generate the fragments of C_42_H_71_O_13_^−^ with *m/z* 783.4974, C_36_H_61_O_8_^−^ with *m/z* 621.4423 and C_30_H_51_O_3_^−^ with *m/z* 459.3872, respectively. The fragment of C_30_H_51_O_3_^−^ with *m/z* 459.3872 is a characteristic fragment of PPD type ginsenosides. Ginsenoside Re, which belongs to PPT type ginsenosides, performed neutral loss of a rhamnose residual of 146.0579 to generate the fragment of C_42_H_71_O_14_^−^ with *m/z* 799.4893, then performed neutral loss of 1 and 2 glucose residuals of 162.0528 to generate the fragments of C_36_H_61_O_9_^−^ with *m/z* 637.4351 and C_30_H_51_O_4_^−^ with *m/z* 475.3795, respectively. The fragment of C_30_H_51_O_4_^−^ with *m/z* 475.3795 is a characteristic fragment of PPT type ginsenosides. The mass defect 0.49221 of ginsenoside Rg1 was selected as the mass defect filtering reference along with the defect tolerance of 100 mDa and the filtering mass range of 600–1250 Da. The fragments of *m/z* 459.3872 and *m/z* 475.3795 in negative ion mode were used as the fragment filtering references along with applying the neutral loss filtering of saccharide residuals.

#### 2.2.5. Identification of Flavonoids in WZYD

Flavonoids possibly exist in both Euodiae Fructus and Jujubae Fructus. Common flavonoids such as rutin, quercetin, spinosin and a few others were detected and listed in Table S1. Flavonoid glycosides usually lost their saccharide groups and generated fragments like their aglycones. For instance, rutin lost a rhamnose residual of 146.0579 and then lost a glucose residual of 162.0528 to generate the fragments of C_21_H_21_O_12_^+^ with *m/z* 465.1017 and C_15_H_11_O_7_^+^ with *m/z* 303.0506 in positive ion mode. It is worth mentioning that these kinds of glycoside compounds can normally use the neutral loss filtering in the identification process. In negative ion mode, the fragment of C_15_H_9_O_7_^−^ with *m/z* 301.0354 showed up by losing a rhamnose and a glucose residual of the glycoside. Meanwhile, the CID exhibited the fragments of C_7_H_3_O_4_^−^ with *m/z* 151.0028, C_6_HO_3_^−^ with *m/z* 121.0394, and C_6_H_3_O_2_^−^ with *m/z* 107.0119, *etc.*, which were all shown in the fragmentation of quercetin, the aglycone of rutin. The fragmentation pattern is shown in Figure S3.

#### 2.2.6. Organic Acids and Other Compounds in WZYD

Organic acids are distributed in most of the herbs, and the origin of organic acids in WZYD might include all four herbs. The fragmentation of the representative compound chlorogenic acid is shown in Figure S4. Chlorogenic acid can be chemically attributed to phenylpropanoid, and it is an ester formed by the esterification of a caffeic acid and a quinic acid. In positive ion mode of fragmentation, it lost the quinic acid and formed the base peak of C_9_H_7_O_3_^+^ with *m/z* 163.0392, and in negative ion mode, it lost the caffeic acid and formed the base peak of C_7_H_11_O_6_^−^ with *m/z* 191.0569. Other identified or predicted compounds included alkaloids, triterpenoid and steroidal saponins, 2,2-dimethylchromenes, lignans, coumarins, terpenoids and a few saccharides. Relative profiles are all listed in Table S1. The qualitative investigation and structural demonstration of the ingredients have provided data for further studies of WZYD. To sum up, using the established strategy, 168 compounds were structurally characterized in a fast and reliable manner. The results clearly indicate the complexity of identifying the herbal compounds.

## 3. Materials and Methods 

### 3.1. Chemicals and Materials

HPLC grade acetonitrile, methanol, formic acid and water were all purchased from Fisher Scientific (Fair Lawn, NJ, USA). Euodiae Fructus, Zingiberis Rhizoma Recens, Ginseng Radix et Rhizoma and Jujubae Fructus were all purchased from the Tong-Ren-Tang TCM store (Shenyang, China) and authenticated by Ying Jia (School of Chinese Material Medica, Shenyang Pharmaceutical University, Shenyang, China).

### 3.2. Preparation of WZYD

WZYD was prepared with the following procedure based on the original composition and preparation method. 5.6 g of Euodiae Fructus, 7.6 g of Zingiberis Rhizoma Recens, 3.8 g of Ginseng Radix et Rhizoma and 3.0 g of Jujubae Fructus were boiled together in 200 mL water for 1 h and then filtered through the filter paper. The herbs were extracted twice. The extracts were then combined and concentrated under reduced pressure to 0.8 g crude drug per milliliter followed by diluting to 0.1 g crude drug per milliliter in water, and filtered through a 0.22 μm syringe filter twice for final purification.

### 3.3. Apparatus and LC-MS Conditions

The UHPLC-QTOF tandem mass spectrometry system was performed with a Prominence™ LC-20A UFLC XR LC System (Shimadzu, Kyoto, Japan) and an Sciex TripleTOF 4600 Quadrupole Time-of-Flight tandem mass spectrometer equipped with TurboV source and a TurboIonspray interface (Sciex, Redwood City, CA, USA). All the operations, the acquisition were controlled by the Analyst TF software (version 1.6, Sciex). The chromatographic separation was performed on a Phenomenex Kinetex C18 (100 mm × 4.6 mm i.d., particle size 2.6 µm, Phenomenex, Torrance, CA, USA) column at 30 °C. Analysis was completed with a gradient elution of 0.1% formic acid in water (A)—0.1% formic acid in acetonitrile (B) within 35.0 min. The gradient program was 98% A → 55% A at 0–27.0 min; 55% A → 25% A at 27.0–30.0 min; 25% A → 10% A at 30.0–31.0 min; 98% A at 31.0–35.0 min at a flow rate of 0.5 mL·min^−1^ with a sample injection volume of 2.0 μL at 4 °C. The MS method consisted of a TOF MS full scan and IDA-fragmentation scan with both positive and negative electrospray ionization. Source parameters were as follows: temperature, 600 °C; ion spray voltage, 5500 V (positive ion mode)/−4500 V (negative ion mode); nebulizer gas (Gas 1), 60 psi; heater gas (Gas 2), 60 psi; curtain gas, 35 psi; declustering potential, 60 V (positive ion mode)/−60 V (negative ion mode); and collision energy, 10 V (positive ion mode)/−10 V (negative ion mode). In IDA, dynamic background subtraction (DBS) was activated for best IDA coverage; spectra exceeding 50 cps were selected for 8 dependent CID fragmentation scans; isotopes within 4 Da were excluded; mass tolerance was 50 mDa; and collision energy was set to 45 V (positive ion mode)/−45 V (negative ion mode) with a collision energy spread of 15 V. One experimental period of 840 ms consisted of 9 experiments, the first of which was the TOF MS scan of 150 ms, and the other 8 were the CID fragmentation scans of 80 ms each. Nitrogen was kept as nebulizer and auxiliary gas. The TOF MS full scan was operated with the mass range of *m/z* 100–1500 Da and the CID fragmentation scan was operated with the mass range of *m/z* 80–1250 Da.

### 3.4. MS Data Processing

The LC-MS data files were further processed with multiple data processing approach using the Sciex PeakView^®^ (version 2.2) software. For further confirmation of the structures, all data were processed using the MasterView™ (version 1.1) software. TOF-MS information was used to screen and identify targeted ingredients in WZYD. The identification was based on accurate mass of quasi-molecular ion, isotopic pattern and MS/MS fragmentation pattern. Samples were compared against control (blank) sample to further identify unexpected ingredients. The ions which were present in the WZYD sample and absent in the control sample were extracted and analyzed using the in-built formula finder module in the MasterView software.

#### Mass Defect and Fragment Filtering Approach and Formula Prediction

Mass defect is the difference between the nominal mass and the monoisotopic mass of an ion in mass spectrometry. Since high accuracy and high resolution can be acquired by the TOF mass analyzer, MDF was used to facilitate the detection of characteristic ingredients by an in-built module in the PeakView Software. Phytocompounds in the same family normally share a similar core substructure while substitutions and other reactions made them variation. The substituents usually have relatively minor and defined changes in the mass detect of the core substructure. As a matter of fact, the mass defect profile of the analogues typically changed within a limited range. Therefore, the procedure of the MDF was to set a filtering reference in the first place based on all the structures of the ingredients reported. Moreover, a mass range of the MDF filter also needed to be established based on the potential compounds [14]. After the filtering was applied to the TIC, the heterogeneous ions would be eliminated and target ions headed above surface.

Theoretically, the same fragment ions can be found in the same types of compounds since compounds with similar structures normally have similar fragmentation patterns, and besides, neutral loss usually happens in glycosides by losing saccharide residuals. Consequently, fragment and neutral loss filter were applied for further prediction and confirmation by an in-built module in the PeakView Software.

## 4. Conclusions

One of the most challenging and important tasks in comprehensive qualitative analysis of herbal prescriptions is to enable rapid and accurate performance analysis. In this study, the established strategy using high resolution mass spectrometry along with MDF and a fragment filtering approach was successfully applied to rapidly identify the characteristic structural analogues from a complex herbal extract. In conclusion, a total of 168 compounds were characterized, including 23 evodiamines and their analogous alkaloids, 12 limonoids, 17 gingerols, 38 ginsenosides, 15 flavonoids, 16 organic acids, 14 alkaloids, 5 saponins, 3 2,2-dimethylchromenes and 25 other compounds. Fragmentation pathways of the representative compounds were specifically illustrated to reveal the fragmentation pattern of each compound family. Conventional screening coupled with MDF and a fragment filtering approach enabled the original data to be analyzed more quickly and accurately by reducing potential interference from matrix ions. This study was the first report on complete, comprehensive qualitative analysis of WZYD. Moreover, the strategy presented here could open new paths for similar studies on other herbal drugs and prescriptions.

## Supplementary Materials

Supplementary materials can be accessed at: http://www.mdpi.com/1420-3049/21/5/664/s1.

## Abbreviations

The following abbreviations are used in this manuscript:

BPC	Base peak chromatogram
CID	Collision induced dissociation
DBS	Dynamic background subtraction
IDA	Information dependent acquisition
LC	Liquid chromatography
MDF	Mass defect filtering
MS	Mass spectrometry
PPD	Protopanaxadiol
PPT	Protopanaxatriol
QTOF	Quadrupole time-of-flight
TCC	Total compound chromatogram
TCM	Traditional Chinese Medicine
TIC	Total ion chromatogram
WZYD	Wu-Zhu-Yu decoction
XIC	Extracted ion chromatogram
