# Supplementary material for: Comprehensive Qualitative Ingredient Profiling of Chinese Herbal Formula Wu-Zhu-Yu Decoction via a Mass Defect and Fragment Filtering Approach Using High Resolution Mass Spectrometry
Source: Molecules. 2016 May 19;21(5):664. doi: 10.3390/molecules21050664 (PMC6273025; doi:10.3390/molecules21050664)
Supplement: Supplementary file 1 [file molecules-21-00664-s001.pdf]

Supplementary Materials: Comprehensive Ingredients Qualitative Profiling of Chinese Herbal Formula Wu-Zhu-Yu Decoction Based on Mass Defect and Fragment Filtering Approach Using High Resolution Mass Spectrometry

Huarong Xu, Huibin Niu, Bosai He, Chang Cui, Qing Li and Kaishun Bi

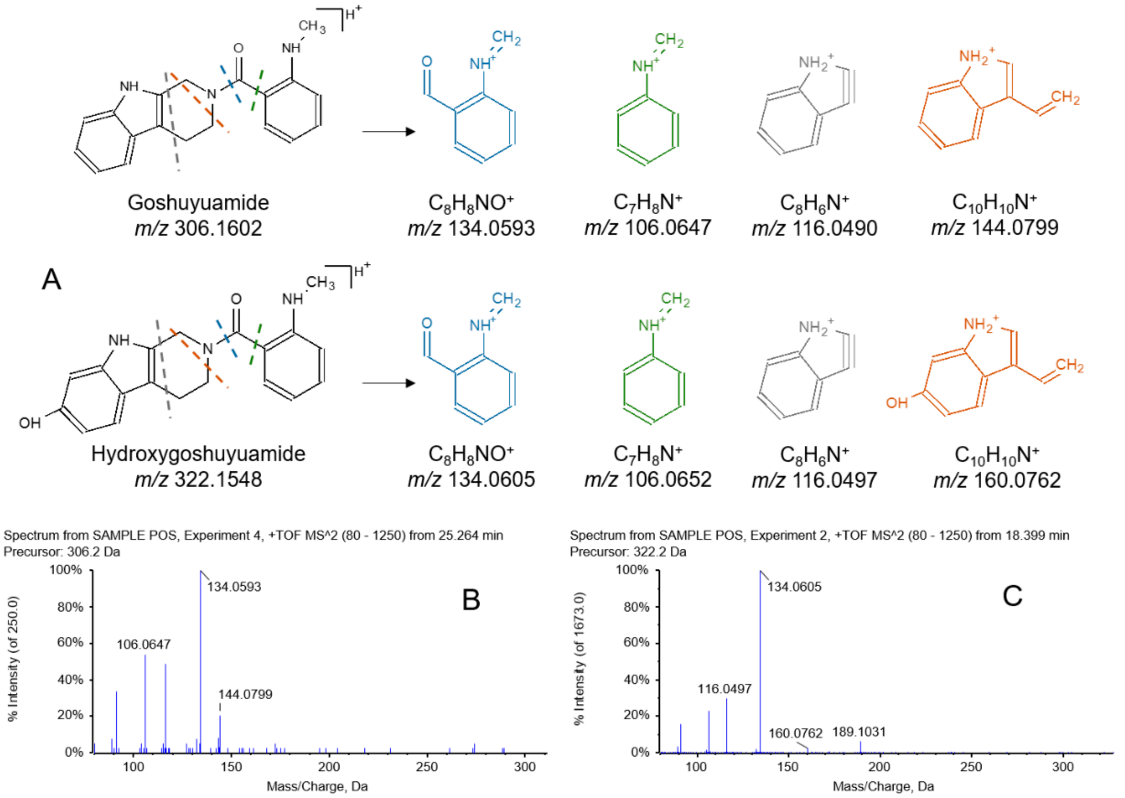

Figure S1. Fragmentation pattern of goshuyuamide and hydroxygoshuyuamide. (**A**) Representative fragments of goshuyuamide and hydroxygoshuyuamide; (**B**) product ion MS/MS spectrum of goshuyuamide; (**C**) product ion MS/MS spectrum of hydroxygoshuyuamide.

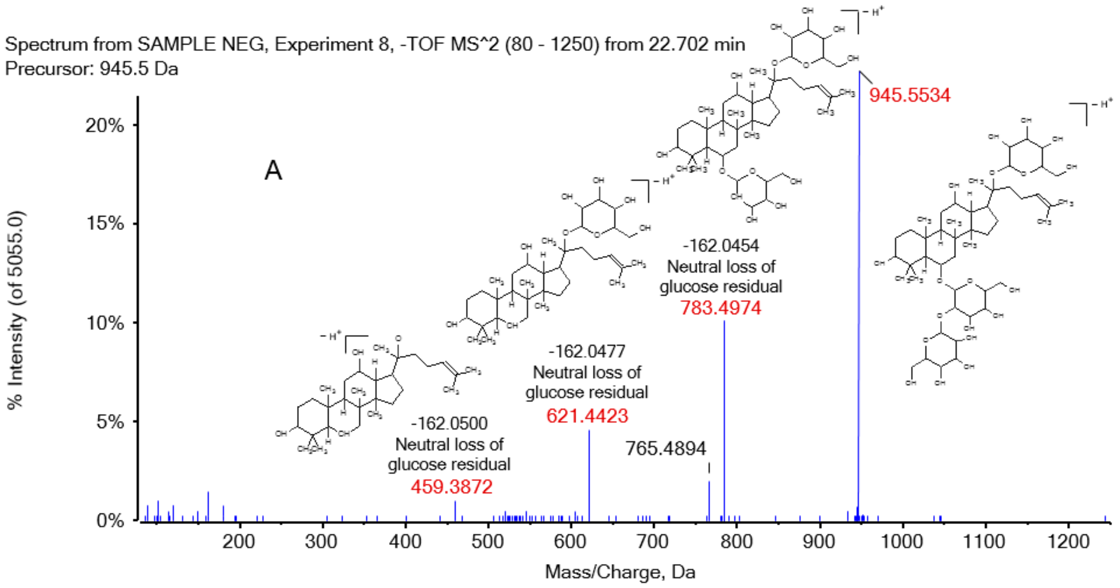

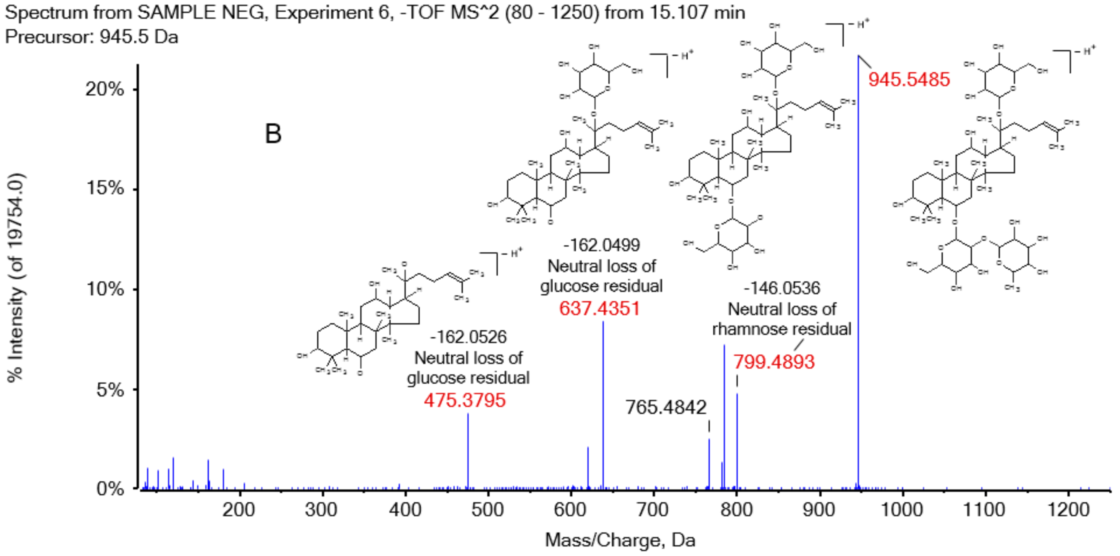

Figure S2. (A) Fragmentation pathways and product ion MS/MS spectrums of (**A**) PPD type ginsenoside Rd and (**B**) PPT type ginsenoside Re.

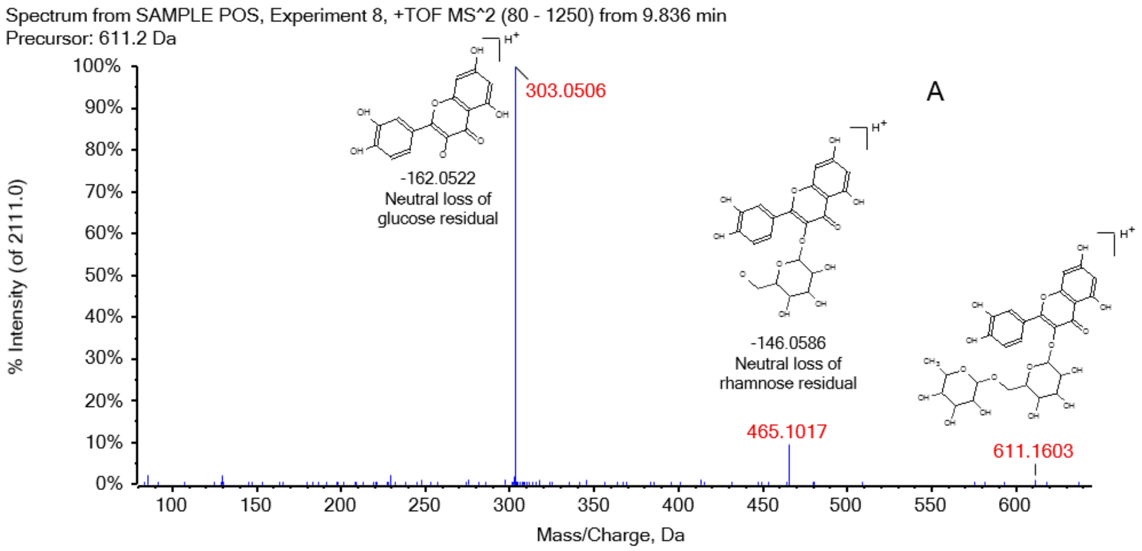

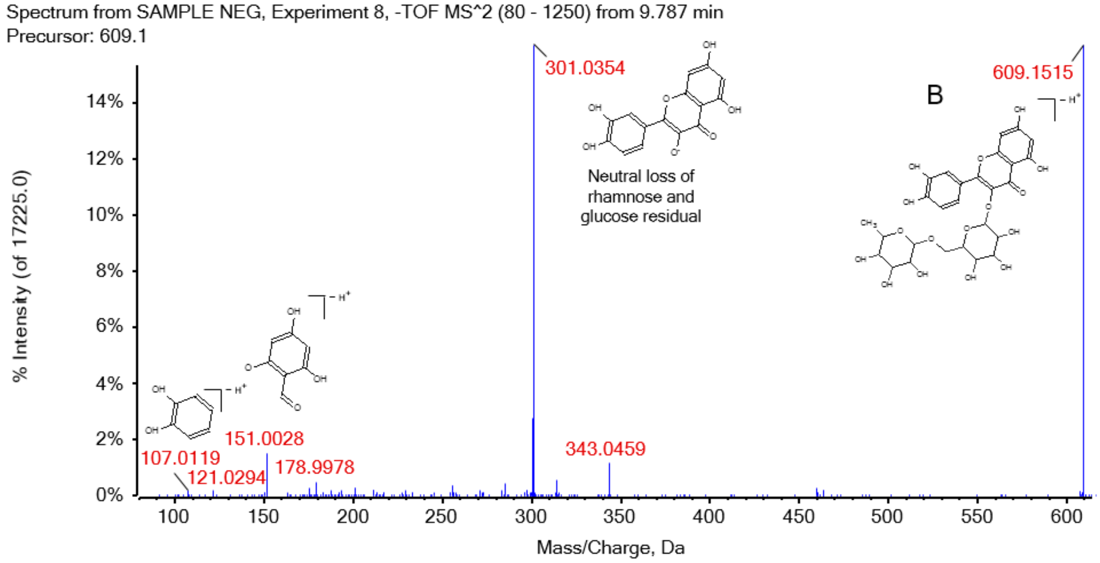

Figure S3. Fragmentation pathways and product ion MS/MS spectrums of rutin in (**A**) positive and (**B**) negative ion mode.

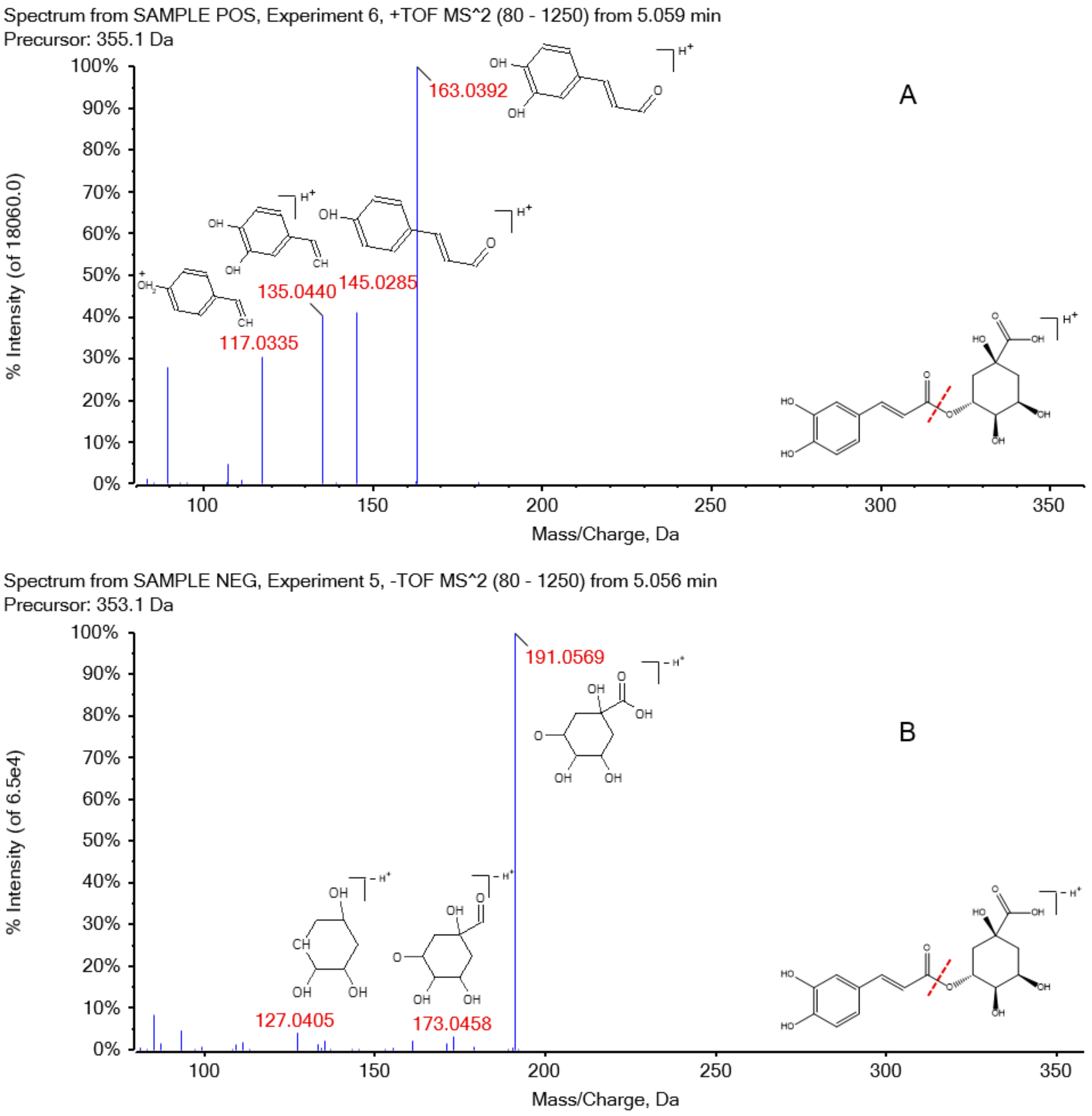

Figure S4. Fragmentation pathways and product ion MS/MS spectrums of chlorogenic acid in (**A**) positive and (**B**) negative ion mode.

Table S1. Characterization of the ingredients in Wu-Zhu-Yu decoction by LC high resolution MS.

No.	Compound	Formula	Ion Adduction	Theoretical *m/z* (Da)	Measured *m/z* (Da)	Error/ ppm	t_R/min	Product Ions (*m/z*, Da)
				**Evodiamine and its analogous alkaloids**				
1	Dihydroxyevodiamine	$C_{19}H_{17}N_3O_3$	$[M + H]^+$	336.13427	336.13458	0.9	7.57	134.0599, 161.0711, 176.0707, 148.0394, 146.0597, 120.0446, 160.0636, 106.0648, 162.0541, 130.0288
2	Hydroevoprenine	$C_{20}H_{23}NO_4$	$[M + H]^+$	342.16998	342.17001	0.1	7.85	282.0885, 222.0674, 235.0752, 311.1265, 310.1427, 207.0804, 236.0832, 252.0779, 256.1072, 270.0882, 242.0948, 292.1337, 308.1300
3	Evoprenine	$C_{20}H_{21}NO_4$	$[M + H]^+$	340.15433	340.15384	−1.5	8.16	220.0520, 189.0699, 217.0636, 308.1249, 276.1055, 196.0963, 295.0963, 189.0820, 266.1210, 282.1120, 290.1170, 310.1183324.1239
4	Methyl-Dihydroevoprenine	$C_{21}H_{27}NO_4$	$[M + H]^+$	358.20129	358.20147	0.5	9.32	160.0757, 282.1254, 298.1201, 312.1594, 253.1221, 221.0967, 286.1197, 107.0491, 329.1747, 300.1353, 223.1118, 136.0515, 252.1141, 240.1019, 268.1104, 326.1748
5	Hydroxyevodiamine	$C_{19}H_{17}N_3O_2$	$[M + H]^+$	320.13935	320.13908	−0.9	9.35	134.0584, 161.0703, 160.0759, 91.0553
6	Methyl-Hydroevoprenine	$C_{21}H_{25}NO_4$	$[M + H]^+$	356.18564	356.18563	0.0	10.08	236.0841, 249.0911, 221.0968, 298.1202, 282.1272, 266.0932, 252.0785, 310.1188
7	Dihydroxyrutaecarpine	$C_{18}H_{13}N_3O_3$	$[M + H]^+$	320.10297	320.10276	−0.6	11.14	169.0403, 302.0925, 134.0603, 114.0337, 274.1102, 128.0471, 159.0540, 247.0883, 104.0481, 156.0448, 169.0233, 276.1154,
8	Methyl-Hydroxyevodiamine	$C_{20}H_{19}N_3O_2$	$[M + H]^+$	334.15500	334.15494	−0.2	12.21	134.0592, 161.0711, 174.0915, 130.0648, 91.0540,
9	Dehydroevodiamine	$C_{19}H_{15}N_3O$	$[M + H]^+$	302.12879	302.12855	−0.8	12.44	286.0975, 258.1026, 272.0824, 167.0608, 259.0870, 134.0595, 132.0445, 106.0651, 231.0916
10	Methyl-hydroxyrutaecarpine	$C_{19}H_{15}N_3O_2$	$[M + H]^+$	318.12370	318.12374	0.1	12.68	285.0898, 256.0875, 300.1134, 284.0824, 274.1337, 230.0847
11	Methyl-dihydrorutaecarpine	$C_{19}H_{17}N_3O$	$[M + H]^+$	304.14444	304.14409	−1.1	12.88	144.0807, 161.0708, 120.0439, 92.0487, 128.0490, 134.0592, 116.0499, 142.0647, 118.0645, 145.0389
12	Ethyl-hydroxyrutaecarpine	$C_{20}H_{17}N_3O_2$	$[M + H]^+$	332.13935	332.13902	−1.0	13.34	302.0915, 274.0967, 316.1072, 317.1147, 289.1200, 288.1124, 272.0816, 247.0863, 197.0706, 300.1129, 262.0958, 258.0779, 130.0649, 245.0715, 233.0711, 116.0484, 186.0903, 230.0833, 237.1015
13	Evodianinine	$C_{19}H_{13}N_3O$	$[M + H]^+$	300.11314	300.11323	0.3	13.66	285.0896, 257.0950, 256.0873, 300.1137, 229.0763, 284.0822, 231.0797, 155.0609, 167.0609, 270.1031, 128.0502
14	Wuchuyuamide I	$C_{19}H_{17}N_3O_4$	$[M + H]^+$	352.12918	352.12889	−0.8	14.18	189.0656, 103.0537, 159.0544, 177.0661, 104.0486, 132.0446, 120.0442
15	Evolitrine	$C_{13}H_{11}NO_3$	$[M + H]^+$	230.08117	230.08125	0.3	17.39	200.0354, 186.0546, 214.0503, 172.0396
16	Hydroxygoshuyuamide	$C_{19}H_{19}N_3O_2$	$[M + H]^+$	322.15500	322.15475	−0.8	18.40	134.0593, 106.0647, 116.0490, 160.0762, 189.1031
17	Hydroxyrutaecarpine	$C_{18}H_{13}N_3O_2$	$[M + H]^+$	304.10805	304.10823	0.6	19.34	286.0979, 258.1032, 140.0494, 274.0962, 259.0883, 120.0446, 128.0510, 147.0581, 183.0581, 228.0702
18	Evodiamide	$C_{19}H_{21}N_3O$	$[M + H]^+$	308.17574	308.17576	0.1	22.47	134.0598, 116.0495, 106.0649, 144.0805, 132.0441, 105.0338

Table S1. *Cont.*

No.	Compound	Formula	Ion Adduction	Theoretical *m/z* (Da)	Measured *m/z* (Da)	Error/ ppm	t_R/min	Product Ions (*m/z*, Da)
19	Evodiamine	$C_{19}H_{17}N_3O$	$[M + H]^+$	304.14444	304.14454	0.3	22.75	134.0602, 161.0710, 171.0918, 144.0808, 116.0494, 106.0651
20	Rutaecarpine	$C_{18}H_{13}N_3O$	$[M + H]^+$	288.11314	288.11340	0.9	23.59	273.0903, 169.0767, 243.0922, 272.0820, 167.0607, 145.0404, 120.0447, 219.0925, 116.0496, 144.0816, 231.0931, 143.0605, 92.0499,
21	Goshuyuamide	$C_{19}H_{19}N_3O$	$[M + H]^+$	306.16009	306.16017	0.3	25.27	134.0593, 106.0647, 116.0490, 144.0799, 132.0451
22	Evocarpine	$C_{23}H_{33}NO$	$[M + H]^+$	340.26349	340.26324	−0.7	31.89	186.0914, 200.1061, 158.0602, 242.1531, 172.0762, 228.1386, 256.1693, 214.1218, 198.1272, 132.0563, 160.0739, 296.1997, 144.0445, 167.0739, 270.1841, 312.2319
23	Dihydroevocarpine	$C_{23}H_{35}NO$	$[M + H]^+$	342.27914	342.27856	−1.7	32.61	186.0920, 200.1069, 132.0565, 158.0611, 144.0802, 172.0769, 228.1417, 160.0751, 284.1982, 104.0619, 214.1289, 242.1540, 256.1695
**Limonoids**								
24	Evodinone	$C_{26}H_{30}O_9$	$[M + H]^+$	487.19626	487.19640	0.3	8.09	441.1930, 469.1856, 197.0974, 95.0131, 227.1060, 321.1474, 81.0331, 259.0973, 205.0526, 219.0618, 401.1558, 289.1198, 279.1374, 209.0956, 163.1426, 265.1224, 261.1272
25	Rutaevine	$C_{26}H_{30}O_9$	$[M + H]^+$	487.19626	487.19598	−0.6	12.16	121.0280, 95.0126, 197.0964, 179.0857, 193.1002, 221.0968, 209.0951, 105.0697, 135.0801, 167.0845
			$[M - H]^-$	485.18171	485.18297	2.6	12.14	345.1705, 129.0555, 273.1126, 261.1124, 95.0138, 227.1069, 111.0448
26	Rutaevine Acetate	$C_{28}H_{32}O_{10}$	$[M + H]^+$	529.20682	529.20619	−1.2	13.21	451.1752, 393.1335, 175.0761, 425.1952, 105.0706, 95.0134,205.0505, 349.1436, 133.0653, 321.1489,277.1232,365.1746,261.1275, 441.1903,121.0653,469.1846, 289.1227
27	12α-Hydroxyevodol	$C_{26}H_{28}O_{10}$	$[M + H]^+$	501.17552	501.17475	−1.6	17.44	443.2064, 95.0121, 211.1117, 329.1496, 425.1923, 105.0696, 209.0960, 307.1692, 427.2089, 383.1832, 133.0638, 279.1358, 429.1895, 411.1787, 331.1676
			$[M - H]^-$	499.16097	499.16131	0.7	17.42	437.16118, 439.1432, 411.1450, 135.0442, 123.0454, 149.0602, 163.0407, 209.0957, 239.1066, 347.1664, 171.0828, 197.0973, 211.0748, 251.1045, 263.0925, 277.1254, 349.1398
28	Limonoic acid A-ring lactone	$C_{26}H_{32}O_9$	$[M + H]^+$	489.21191	489.21149	−0.9	17.85	443.2064, 277.1226, 211.1117, 425.1923, 223.1093, 305.1519, 251.1049, 169.1004, 183.0795, 213.0904, 237.0900, 241.1227
			$[M - H]^-$	487.19736	487.19890	3.2	17.84	337.1446, 457.1877, 293.1176, 413.1967, 295.1334, 241.1221, 309.1485, 351.1956, 355.1565, 385.2005, 213.1271, 317.1750, 395.1887, 133.0658, 159.0802, 197.0957
29	Graucin A	$C_{26}H_{30}O_{10}$	$[M + H]^+$	503.19117	503.19055	−1.2	17.98	105.0700, 365.1400, 395.1464, 95.0134, 323.1277, 205.0503, 265.1206, 277.1232, 353.1756, 503.1917, 137.0601, 151.0750, 209.0814, 221.0807
			$[M - H]^-$	501.17662	501.17786	2.5	17.96	411.1460, 409.1668, 471.1683, 427.1774, 393.1347
30	Obacunone	$C_{26}H_{30}O_7$	$[M + H]^+$	455.20643	455.20594	−1.1	18.25	235.1099, 171.0831, 195.1191, 207.1154, 247.0953

Table S1. *Cont.*

No.	Compound	Formula	Ion Adduction	Theoretical *m*/*z* (Da)	Measured *m*/*z* (Da)	Error/ ppm	t_R/min	Product Ions (*m*/*z*, Da)
31	12*α*-Hydroxylimonin	$C_{26}H_{30}O_9$	$[M + H]^+$	487.19626	487.19590	−0.7	18.72	441.1903, 469.1856, 105.0698, 443.2077 ,277.1217, 383.1476, 197.0974, 365.1376
			$[M - H]^-$	485.18171	485.18330	3.3	18.69	123.0457, 485.1845, 425.1622, 423.1828, 347.1296, 379.1563, 307.1339, 405.1720, 241.1235, 211.1131, 251.1083, 223.1131, 105.0347,111.0453
32	Limonin	$C_{26}H_{30}O_8$	$[M + H]^+$	471.20134	471.20104	−0.6	20.01	425.1942, 213.0913, 95.0136, 105.0703, 367.1890, 133.0653, 453.1889, 205.0495, 411.1803
			$[M - H]^-$	469.18679	469.18729	1.1	19.96	381.2103, 223.1128, 451.1834, 135.0819, 107.0529, 249.1274, 395.1884, 197.0989, 209.0991, 259.1006, 263.1448, 321.1486
33	Evodol	$C_{26}H_{28}O_9$	$[M + H]^+$	485.18061	485.18080	0.4	21.05	381.1705, 439.1774, 105.0699, 133.0650, 425.1600, 137.0942, 363.1599
			$[M - H]^-$	483.16606	483.16698	1.9	20.96	421.1679, 423.1467, 395.1513, 135.0451, 259.0979, 377.1400, 163.0402, 403.1562, 375.1612, 333.1495, 349.1445, 263.1437
34	Jangomolide	$C_{26}H_{28}O_8$	$[M + H]^+$	469.18569	469.18512	−1.2	21.90	95.0124, 221.0952, 165.0701, 195.0815, 219.0805, 133.0645, 169.0661, 179.0865, 289.1233, 423.1824，125.0599，195.1191, 211.0769, 233.0943, 249.1121, 117.0709, 135.0807, 107.0483, 149.0595, 205.0517, 209.0834, 231.0632, 243.0782, 451.1743
			$[M - H]^-$	467.17114	467.17260	3.1	21.80	423.1834, 365.1422, 207.0649, 349.1106, 249.1300, 135.0800, 147.0427, 193.1015, 221.0968, 263.1436, 83.0495, 111.0438, 121.0264, 133.0679, 217.0472, 223.1079, 259.0983, 421.16332
35	6*α*-acetoxy-5-epilimonin	$C_{28}H_{32}O_{10}$	$[M + H]^+$	529.20682	529.20667	−0.3	22.69	393.1341, 205.0496, 349.1441, 365.1730, 95.0126, 117.0677, 173.0956, 4233.1795
			$[M - H]^-$	527.19227	527.19415	3.6	22.58	485.1845, 339.1612, 441.1937, 409.1297, 305.1042, 259.0987, 149.0608, 267.1396, 439.1780
**Gingerols**								
36	4-Gingerol	$C_{15}H_{22}O_4$	$[M + H]^+$	267.15909	267.15935	1.0	16.30	-
			$[M - H]^-$	265.14453	265.14449	−0.2	16.32	-
37	Gingerenone A	$C_{21}H_{24}O_5$	$[M + H]^+$	357.16965	357.16932	−0.9	16.36	-
38	Hexahydrocurcumin	$C_{21}H_{26}O_6$	$[M + H]^+$	375.18022	375.17990	−0.9	16.36	137.0602, 103,0548, 177.0912, 115.0570, 163.0756, 179.0707, 165.0982
			$[M - H]^-$	373.16566	373.16598	0.8	16.36	179.0709, 121.0287, 135.0451, 163.0402, 193.0889
39	6-Gingediacetate	$C_{21}H_{32}O_6$	$[M + H]^+$	381.22717	381.22741	0.6	17.51	-
40	7-Paradol	$C_{18}H_{28}O_3$	$[M + H]^+$	293.21112	293.21078	−1.2	18.08	91.0551, 131.0851
41	Methyl-6-Shogaol	$C_{18}H_{26}O_3$	$[M + H]^+$	291.19547	291.19554	0.2	21.89	93.0692, 107.0510, 115.0545, 135.0772
			$[M - H]^-$	289.18092	289.18082	−0.3	21.80	-
42	7-Gingerol	$C_{18}H_{28}O_4$	$[M + H]^+$	309.20604	309.20576	−0.9	21.89	91.0542, 105.0705, 107.0490, 95.0861, 97.1019, 145.0999, 121.0645, 125.0949, 131.0857, 135.0871
			$[M - H]^-$	307.19148	307.19167	0.6	21.79	121.0662, 125.0994, 184.1241, 235.1337, 97.0646, 137.0939, 211.1326

Table S1. *Cont.*

No.	Compound	Formula	Ion Adduction	Theoretical *m*/*z* (Da)	Measured *m*/*z* (Da)	Error/ ppm	t_R/min	Product Ions (*m*/*z*, Da)
43	6-Gingerol	$C_{17}H_{26}O_4$	$[M + H]^+$	295.19039	295.19063	0.8	22.36	137.0694, 91.0537, 145.0652, 177.0921, 179.0716,
			$[M − H]^-$	293.17583	293.17589	0.2	22.28	99.0816, 135.0461, 177.0585
44	6-Shogaol	$C_{17}H_{24}O_3$	$[M + H]^+$	277.17982	277.17999	0.6	22.36	137.0595, 177.0906, 91.0537, 161.0594, 175.0767
45	Dehydro-6-Gingerol	$C_{17}H_{24}O_4$	$[M + H]^+$	293.17474	293.17468	−0.2	22.63	89.0376, 149.0570, 177.0549
			$[M − H]^-$	291.16018	291.16045	0.9	22.54	-
46	8-Shogaol	$C_{19}H_{28}O_3$	$[M + H]^+$	305.21112	305.21137	0.8	28.15	91.0543, 137.0618, 177.0861
47	6-Gingerdiol	$C_{17}H_{28}O_4$	$[M + H]^+$	297.20604	297.20596	−0.3	29.74	91.0554, 93.0673, 121.1000
			$[M − H]^-$	295.19148	295.19175	0.9	29.73	-
48	Dehydro-8-Gingerol	$C_{19}H_{28}O_4$	$[M + H]^+$	321.20604	321.20576	−0.9	30.22	137.0610, 103.0529
49	Dehydro-6-Gingerdione	$C_{17}H_{22}O_4$	$[M + H]^+$	291.15909	291.15917	0.3	30.36	-
			$[M − H]^-$	289.14453	289.14446	−0.2	30.36	-
50	8-Gingerol	$C_{19}H_{30}O_4$	$[M + H]^+$	323.22169	323.22137	−1.0	30.71	93.0673, 121.1000, 187.1449, 207.0341
			$[M − H]^-$	321.20713	321.20742	0.9	30.71	80.9659, 101.0606
51	10-Shogaol	$C_{21}H_{32}O_3$	$[M + H]^+$	333.24242	333.24211	−0.9	30.91	93.0673, 95.0838, 121.1000
52	8-Gingerdiol	$C_{19}H_{32}O_4$	$[M + H]^+$	325.23734	325.23672	−1.9	31.27	145.0667, 95.0853, 119.0868, 107.0851, 91.0533, 93.0685
			$[M − H]^-$	323.22278	323.22331	1.6	31.28	101.0604, 239.1668
**Ginsenosides**								
53	Notoginsenoside R6	$C_{48}H_{82}O_{19}$	$[M − H]^-$	961.53775	961.53878	1.1	13.56	799.4880, 179.0537, 781.4785, 89.0239, 637.4348, 119.0348, 323.0973, 619.4241, 101.0230, 161.0443
			$[M + Na]^+$	985.53425	985.53282	−1.5	13.54	-
54	Floralginsenoside A	$C_{42}H_{72}O_{16}$	$[M − H]^-$	831.47476	831.47681	2.5	13.67	785.4719, 653.4309
55	20-Glucoginsenoside Rf	$C_{48}H_{82}O_{19}$	$[M − H]^-$	961.53775	961.54023	2.6	13.97	799.4890, 637.4367, 89.0245, 781.4777, 475.3808, 619.4249, 161.0451, 179.0549, 101.0241, 119.0340
			$[M + Na]^+$	985.53425	985.53330	−1.0	13.94	-
56	Notoginsenoside R9	$C_{36}H_{62}O_{10}$	$[M + HCOOH-H]^-$	699.43140	699.43444	4.3	14.04	491.3771, 653.4142, 553.3523
			$[M + Na]^+$	677.42352	677.42103	−3.7	14.02	-
57	Ginsenoside Ib	$C_{42}H_{72}O_{14}$	$[M − H]^-$	799.48493	799.48799	3.8	14.24	-
58	Notoginsenoside R3	$C_{48}H_{82}O_{19}$	$[M − H]^-$	961.53775	961.54080	3.2	14.34	221.0668, 637.4357, 179.0560, 781.4793, 323.0976, 799.4862, 101.0245, 119.0358, 161.0488, 263.0779
			$[M + Na]^+$	985.53425	985.53235	−1.9	14.31	-
59	Notoginsenoside R1	$C_{47}H_{80}O_{18}$	$[M − H]^-$	931.52719	931.53059	3.6	14.39	637.4375, 799.4942, 475.3838, 619.4303, 161.0435, 179.0549, 769.4796, 751.4667, 119.0347, 89.0242
			$[M + Na]^+$	955.52369	955.52206	−1.7	14.36	775.4593

Table S1. *Cont.*

No.	Compound	Formula	Ion Adduction	Theoretical *m*/*z* (Da)	Measured *m*/*z* (Da)	Error/ ppm	t_R/min	Product Ions (*m*/*z*, Da)
60	Notoginsenoside N	$C_{48}H_{82}O_{19}$	[M − H]⁻	961.53775	961.54035	2.7	14.64	799.4898, 637.4332, 179.0555, 781.4790, 89.0248, 119.0359, 475.3807, 143.0354, 619.4229, 161.0483
			[M + Na]⁺	985.53425	985.53328	−1.0	14.60	805.4686
61	Notoginsenoside M	$C_{48}H_{82}O_{19}$	[M − H]⁻	961.53775	961.54135	3.7	14.87	799.4919, 637.4403, 161.0454, 323.0993, 101.0233, 143.0365, 203.0550
			[M + Na]⁺	985.53425	985.53521	1.0	14.85	643.4192
62	Ginsenoside Re	$C_{48}H_{82}O_{18}$	[M − H]⁻	945.54284	945.54636	3.7	15.10	637.4351, 783.4943, 799.4893, 475.3795, 765.4842, 619.4253, 119.0341, 161.0451, 781.4805, 89.0239
			[M + Na]⁺	969.53934	969.53839	−1.0	15.06	-
63	Ginsenoside Rg1	$C_{42}H_{72}O_{14}$	[M − H]⁻	799.48493	799.48812	4.0	15.17	-
			[M + Na]⁺	823.48143	823.48071	−0.9	15.13	643.4171, 463.3548, 481.3610
64	Vina-ginsenoside R1	$C_{44}H_{74}O_{15}$	[M − H]⁻	841.49550	841.49746	2.3	15.34	781.4756, 637.4349, 619.4279, 475.3831, 799.4927, 89.0260, 113.0297, 119.0334, 161.0530, 221.0675
65	Ginsenoside F3	$C_{41}H_{70}O_{13}$	[M + HCOOH-H]⁻	815.47875	815.48179	3.7	17.17	637.4353, 475.3791
66	Pseudoginsenoside F11	$C_{42}H_{72}O_{14}$	[M − H]⁻	799.48493	799.48781	3.6	18.67	475.3815, 637.4374, 101.0243, 161.0455, 89.0248, 119.0346, 221.0671, 179.0564, 143.0351, 457.3711
			[M + Na]⁺	823.48143	823.48134	−0.1	18.70	661.4309
67	Ginsenoside Rf	$C_{42}H_{72}O_{14}$	[M − H]⁻	799.48493	799.48781	3.6	19.05	-
			[M + Na]⁺	823.48143	823.47991	−1.8	19.09	-
68	Ginsenoside F5	$C_{41}H_{70}O_{13}$	[M − H]⁻	769.47437	769.47703	3.5	19.33	475.3812, 637.4363, 101.0245, 161.0452, 391.2862, 113.0238, 89.0249, 119.0334, 131.0350, 457.3697
			[M + Na]⁺	793.47086	793.46984	−1.3	19.36	673.4285, 481.3649
69	Ginsenoside Ro	$C_{48}H_{76}O_{19}$	[M − H]⁻	955.49080	955.49368	3.0	19.75	-
			[M + Na]⁺	979.48730	979.48644	−0.9	19.81	641.4019, 623.3902, 817.4336, 185.0413, 393.35525, 439.3506
70	S-Ginsenoside Rg2	$C_{42}H_{72}O_{13}$	[M − H]⁻	783.49002	783.49109	1.4	20.00	475.3804, 637.4360, 101.0240, 619.4231, 163.0611, 143.0354, 159.0298, 391.2842, 89.0243, 205.0720
			[M + Na]⁺	807.48651	807.48711	0.7	20.05	661.4271, 481.3622
71	S-Ginsenoside Rh1	$C_{36}H_{62}O_9$	[M + HCOOH-H]⁻	683.43649	683.43983	4.9	20.21	475.3791, 101.0246, 161.0449, 391.2868, 143.0349, 553.3407, 621.4033, 89.0261, 459.3686, 503.3307
			[M + Na]⁺	661.42861	661.42846	−0.2	20.27	481.3677
72	Ginsenoside Rb1	$C_{54}H_{92}O_{23}$	[M − H]⁻	1107.59566	1107.59921	3.2	20.29	945.5509, 783.4960, 179.0567, 621.4407, 221.0667, 323.0995, 765.4845, 119.0353, 89.0251, 101.0252, 161.0470
			[M + Na]⁺	1131.59216	1131.59178	−0.3	20.37	789.4797

Table S1. *Cont.*

No.	Compound	Formula	Ion Adduction	Theoretical *m*/*z* (Da)	Measured *m*/*z* (Da)	Error/ppm	t_R/min	Product Ions (*m*/*z*, Da)
73	R-Ginsenoside Rg2	$C_{42}H_{72}O_{13}$	$[M + Na]^+$	807.48651	807.48589	−0.8	20.44	661.426
74	Ginsenoside Rs2	$C_{55}H_{92}O_{23}$	$[M + HCOOH-H]^-$	1165.60005	1165.60518	4.4	20.59	-
75	R-Ginsenoside Rh1	$C_{36}H_{62}O_9$	$[M + HCOOH-H]^-$	683.43649	683.43981	4.9	20.71	475.3816, 161.0462, 101.0238, 391.2857, 553.3415,89.0226, 621.4071
			$[M + Na]^+$	661.42861	661.42813	−0.7	20.79	481.3632
76	Ginsenoside Ra1/Ra2	$C_{58}H_{98}O_{26}$	$[M − H]^-$	1209.62736	1209.63248	4.2	20.80	1077.5893, 783.4958, 915.5268, 191.0551, 323.0995, 945.5484, 1047.5825, 131.0401, 293.0933, 621.4412
			$[M + Na]^+$	1233.62386	1233.62361	−0.2	20.90	789.4769
77	Ginsenoside Rc	$C_{53}H_{90}O_{22}$	$[M − H]^-$	1077.58510	1077.58888	3.5	20.86	945.5527, 783.4979, 915.5377, 765.4853, 621.4433, 149.0422, 191.0551, 221.0670, 293.0878, 89.0239, 251.0765
			$[M + Na]^+$	1101.58160	1101.58181	0.2	20.95	789.4774
78	Ginsenoside Rb2	$C_{53}H_{90}O_{22}$	$[M − H]^-$	1077.58510	1077.58992	4.5	21.37	945.5527, 915.5414, 783.4978, 765.4860, 149.0459, 293.0886, 191.0562, 89.0242, 621.4446, 101.0252, 221.0667
			$[M + Na]^+$	1101.58160	1101.58129	−0.3	21.48	789.4762
79	Ginsenoside Rb3	$C_{53}H_{90}O_{22}$	$[M + Na]^+$	1101.58160	1101.58083	−0.7	21.61	789.4760
80	Ginsenoside F1	$C_{36}H_{62}O_9$	$[M + HCOOH-H]^-$	683.43649	683.43943	4.3	22.02	475.3791, 161.0408
			$[M + Na]^+$	661.42861	661.42835	−0.4	22.11	481.3655
81	Ginsenoside Rd	$C_{48}H_{82}O_{18}$	$[M − H]^-$	945.54284	945.54551	2.8	22.71	783.4974, 621.4423, 765.4894, 161.0461, 101.0234, 459.3872, 89.0244, 119.0337, 179.0582
			$[M + Na]^+$	969.53934	969.53884	−0.5	22.81	789.4764
82	Ginsenoside Rg6	$C_{42}H_{70}O_{12}$	$[M − H]^-$	765.47945	765.48240	3.8	25.41	619.4249, 101.0235, 161.0453, 103.0408, 205.0747, 457.3709
			$[M + Na]^+$	789.47595	789.47763	2.1	25.50	-
83	Ginsenoside Rk1	$C_{42}H_{70}O_{12}$	$[M − H]^-$	811.48383	811.48784	4.9	25.94	159.0299, 103.0391, 161.0450, 601.4176, 119.0352, 143.0351, 89.0240, 145.0543
			$[M + Na]^+$	789.47595	789.47486	−1.4	26.01	-
84	Ginsenoside Rk3	$C_{36}H_{60}O_8$	$[M + Na]^+$	643.41804	643.41801	0.0	26.24	-
85	Ginsenoside Rg3	$C_{42}H_{72}O_{13}$	$[M − H]^-$	829.49440	829.49792	4.2	28.30	621.4423, 459.3882, 161.0450, 101.0243, 113.0261, 89.0269, 119.0350. 179.0775, 221.0667
			$[M + Na]^+$	807.48651	807.48626	−0.3	28.33	-
86	Ginsenoside F2	$C_{42}H_{72}O_{13}$	$[M − H]^-$	783.49002	783.49161	2.0	28.69	621.4423, 459.3855, 101.0243, 161.0450, 89.0253, 143.0375, 179.0560, 537.3441, 603.4304
			$[M + Na]^+$	807.48651	807.48619	−0.4	28.74	-

Table S1. *Cont.*

No.	Compound	Formula	Ion Adduction	Theoretical *m*/*z* (Da)	Measured *m*/*z* (Da)	Error/ppm	t_R/min	Product Ions (*m*/*z*, Da)
87	Ginsenoside Rs4	$C_{44}H_{72}O_{13}$	[M + HCOOH-H]⁻	853.49440	853.49844	4.7	30.23	-
88	Ginsenoside Rs5	$C_{44}H_{72}O_{13}$	$[M-H]^-$	853.49440	853.49836	4.6	30.38	765.4842, 603.4314, 747.4759, 161.0459, 101.0263, 645.4415, 203.0626
89	Ginsenoside Ic	$C_{42}H_{70}O_{12}$	$[M-H]^-$	811.48383	811.48719	4.1	30.61	161.0474, 603.4313, 101.0238, 143.0346, 89.0260, 119.0348
			$[M+Na]^+$	789.47595	789.47602	0.1	30.60	-
90	Ginsenoside Rg5	$C_{42}H_{70}O_{12}$	$[M-H]^-$	765.47945	765.48204	3.4	30.76	603.4317, 101.0247, 161.0456, 179.0574, 89.0274, 119.0332, 101.0366, 221.0700, 143.0355
			$[M+Na]^+$	789.47595	789.47549	−0.6	30.75	-
	**Flavonoids**							
91	Kaempferol	$C_{15}H_{10}O_6$	$[M+H]^+$	287.05501	287.05476	−0.9	5.50	-
92	Taxifolin 3-glucoside	$C_{21}H_{22}O_{12}$	$[M-H]^-$	465.10385	465.10454	1.5	5.51	285.0415, 125.0241, 303.0524, 177.0211, 275.0569, 151.0039, 217.0513, 189.0556, 121.0310, 195.0290
93	Spinosin 6'''-(E)-p-coumarate	$C_{37}H_{38}O_{17}$	$[M-H]^-$	753.20362	753.20103	−3.4	7.31	-
94	Quercetin 3-O-rhamnoside	$C_{21}H_{20}O_{11}$	$[M-H]^-$	447.09329	447.09433	2.3	8.66	327.0515, 357.0620, 297.0415, 285.0413, 339.0521, 201.0189
95	Trifolin	$C_{21}H_{20}O_{11}$	$[M-H]^-$	447.09329	447.09433	2.3	8.66	327.0515, 357.0620, 297.04115, 339.0521, 193.0156
96	Hyperoside	$C_{21}H_{20}O_{12}$	$[M+H]^+$	465.10275	465.10232	−0.9	9.55	303.0498, 285.0391, 153.0188, 85.0284, 91.0393, 109.0278, 137.0230, 111.0074, 163.0395, 121.0293
97	Rutin	$C_{27}H_{30}O_{16}$	$[M+H]^+$	611.16066	611.16033	−0.5	9.82	303.0506, 465.1017
			$[M-H]^-$	609.14611	609.14867	4.2	9.78	301.0354, 151.0028, 343.0459, 285.0388, 175.0027, 107.0119, 121.0294
98	Afzelin	$C_{21}H_{20}O_{10}$	$[M+H]^+$	433.11292	433.11286	−0.1	9.87	283.0607, 313.0690, 309.0765, 323.0908, 121.0274
			$[M-H]^-$	431.09837	431.09853	0.4	9.83	311.0569, 341.0662, 269.0442, 281.0446, 253.0516, 237.0576, 353.0689
99	Flavaprenin 7,4'-diglucoside	$C_{32}H_{40}O_{15}$	$[M-H]^-$	663.22944	663.23237	4.4	10.01	619.2438, 299.1656, 635.2390, 457.1887, 559.2215, 369.1711, 161.0610, 531.2252, 119.0349, 89.0247
100	Kaempferol 3-O-rutinose	$C_{27}H_{30}O_{15}$	$[M-H]^-$	593.15119	593.15349	3.9	10.12	175.0401, 199.0400, 151.0037, 257.0462, 133.0316, 215.0350, 241.0493, 243.0310, 269.0453, 149.0254
101	Isorhamnetin-3-O-neohesperidoside	$C_{28}H_{32}O_{16}$	$[M+H]^+$	625.17631	625.17546	−1.4	11.23	317.0650, 285.0386, 129.0539, 479.1180, 97.0283, 147.0651, 153.0181, 127.0381, 329.0656, 301.1006
102	Isorhamnetin-3-O-β-D-galactopyranoside	$C_{22}H_{22}O_{12}$	$[M+H]^+$	479.11840	479.11791	−1.0	11.24	317.0660, 285.0394, 153.0181, 97.0289, 145.0486, 127.0397, 139.0393, 151.0392, 271.0610, 91.0394
103	Monoxerutin	$C_{29}H_{34}O_{17}$	$[M+H]^+$	655.18688	655.18559	−2.0	11.39	347.0751, 85.0284, 301.0324, 315.0505, 129.0547, 509.1243, 145.0513, 287.0546, 331.0398, 147.0656
104	Spinosin	$C_{28}H_{32}O_{15}$	$[M+H]^+$	609.18140	609.18082	−0.9	12.13	301.0714, 463.1239
105	Quercetin	$C_{15}H_{10}O_7$	$[M+H]^+$	303.04993	303.04929	−2.1	13.64	-
			[M − H]-	301.03538	301.03546	0.3	13.64	151.0041, 121.0295, 83.0178, 93.0347, 107.0138

Table S1. *Cont.*

No.	Compound	Formula	Ion Adduction	Theoretical *m*/*z* (Da)	Measured *m*/*z* (Da)	Error/ ppm	t_R/min	Product Ions (*m*/*z*, Da)
				**Organic acids**				
106	Glutamyl-glutamic acid	$C_{10}H_{16}N_2O_7$	$[M + H]^+$	277.10303	277.10270	−1.2	1.17	-
			$[M - H]^-$	275.08847	275.08819	−1.0	1.18	-
107	Citric acid	$C_6H_8O_7$	$[M + H]^+$	193.03428	193.03443	0.8	1.25	-
			$[M - H]^-$	191.01973	191.02021	2.5	1.25	87.0098, 111.0096, 85.0304
108	Pyroglutamic acid	$C_5H_7NO_3$	$[M + H]^+$	130.04987	130.05004	1.3	1.28	84.0442
			$[M - H]^-$	128.03532	128.03589	4.5	1.27	82.0292
109	Nicotinic acid	$C_6H_5NO_2$	$[M + H]^+$	124.03930	124.03917	−1.1	1.55	80.0498, 81.0565
			$[M - H]^-$	122.02475	122.02478	0.3	1.51	91.0178, 95.0146, 119.0132
110	o/p-Coumaric acid	$C_9H_8O_3$	$[M + H]^+$	165.05462	165.05466	0.2	1.57	95.0493, 119.0495
			$[M - H]^-$	163.04007	163.04041	2.1	1.52	-
111	Succinic acid	$C_4H_6O_4$	$[M - H]^-$	117.01933	117.01969	3.1	1.61	-
112	Vanillic acid	$C_8H_8O_4$	$[M + H]^+$	169.04954	169.04944	−0.6	3.04	-
			$[M - H]^-$	167.03498	167.03537	2.3	3.05	109.0299, 121.0299, 123.0471, 91.0181, 149.0274
113	Indole-3-acetic acid	$C_{10}H_9NO_2$	$[M + H]^+$	176.07061	176.07073	0.7	3.62	130.0649, 103.0541, 1147.0673, 104.0496, 102.0468, 118.0651, 105.0573
			$[M - H]^-$	174.05605	174.05686	4.6	3.62	174.0562, 145.0531, 156.0454, 92.0497, 114.0353
114	Salicylic acid	$C_7H_6O_3$	$[M - H]^-$	137.02442	137.02492	3.6	4.36	91.0178, 109.0283, 119.0132
115	Chlorogenic acid	$C_{16}H_{18}O_9$	$[M + H]^+$	355.10236	355.10234	−0.1	5.06	163.0392, 145.0285, 135.0440, 117.03355, 89.0384
			$[M - H]^-$	353.08781	353.08835	1.5	5.06	191.0569, 85.0305
116	3-O-Feruloylquinic acid	$C_{17}H_{20}O_9$	$[M + H]^+$	369.11801	369.11818	0.5	7.45	177.0547, 145.0285, 117.0337, 89.0386, 149.0597
			$[M - H]^-$	367.10346	367.10398	1.4	7.43	191.0570, 173.0462, 93.0355, 193.0513, 111.0456
117	Ferulic acid	$C_{10}H_{10}O_4$	$[M + H]^+$	195.06519	195.06531	0.6	8.72	89.0378, 117.0323, 105.0325, 145.0251, 163.0383, 103.0513, 123.0421, 135.0441
			$[M - H]^-$	193.05063	193.05111	2.5	8.69	133.0288
118	Folic acid	$C_{19}H_{19}N_7O_6$	$[M + H]^+$	442.14696	442.14635	−1.4	9.27	280.0943, 83.0485
119	Cinnamic acid	$C_9H_8O_2$	$[M - H]^-$	147.04515	147.04541	1.7	13.60	-
120	Goshuyic acid/ isomer	$C_{14}H_{24}O_2$	$[M - H]^-$	223.17035	223.17065	1.3	30.83	-
121	Oleanolic acid	$C_{30}H_{48}O_3$	$[M - H]^-$	455.35307	455.35238	−1.5	32.33	-
**Alkaloids**								
122	Synephrine	$C_9H_{13}NO_2$	$[M + H]^+$	168.10191	168.10221	1.8	1.52	107.0491, 134.0602
123	Echinopsine	$C_{10}H_9NO$	$[M + H]^+$	160.07569	160.07562	−0.5	2.67	132.0813, 131.0706, 104.0472, 105.0321
124	Kokusaginine	$C_{14}H_{13}NO_4$	$[M + H]^+$	260.09173	260.09208	1.3	5.27	-

Table S1. *Cont.*

No.	Compound	Formula	Ion Adduction	Theoretical *m*/*z* (Da)	Measured *m*/*z* (Da)	Error/ ppm	t_R/min	Product Ions (*m*/*z*, Da)
125	Aposcopolamine	$C_{17}H_{19}NO_3$	$[M + H]^+$	286.14377	286.14401	0.8	7.22	194.0723, 189.0695, 175.0746, 166.0774, 131.0484, 103.0538, 208.0874, 163.0742, 122.0362, 236.0823
126	Norharman	$C_{11}H_8N_2$	$[M + H]^+$	169.07602	169.07615	0.7	7.41	169.0766, 141.0577, 116.0490, 142.0659, 90.0460
127	Stepharine	$C_{18}H_{19}NO_3$	$[M + H]^+$	298.14377	298.14401	0.8	7.67	192.1024, 238.0987, 210.1028, 254.0923, 250.0988, 269.1156, 253.1208, 266.0931, 234.0662, 281.1124
128	Sinomenine	$C_{19}H_{23}NO_4$	$[M + H]^+$	330.16998	330.16980	−0.6	8.57	137.0596, 299.1271, 194.0723, 284.1022, 208.0891, 287.1268, 272.1025, 121.0644, 298.1434,268.1091
129	Asimilobine	$C_{17}H_{17}NO_2$	$[M + H]^+$	268.13321	268.13352	1.2	9.85	192.0923, 178.0779, 236.0841, 251.1065, 220.0915
130	Isoplatydesmine	$C_{15}H_{17}NO_3$	$[M + H]^+$	260.12812	260.12849	1.4	10.18	184.0757, 200.1074, 242.1182, 158.0611, 172.0766, 132.0571, 104.0479, 103.0541, 127.0547, 157.0875, 176.0711
131	N-Methylasimilobine	$C_{18}H_{19}NO_2$	$[M + H]^+$	282.14886	282.14886	0.0	13.48	234.1039, 250.0795, 192.0979, 236.0839, 206.0734, 282.1494
132	Skimmianine	$C_{14}H_{13}NO_4$	$[M + H]^+$	260.09173	260.09178	0.2	17.67	230.0458, 216.0656, 174.0566, 212.0349, 244.0631, 198.0535, 228.0306, 260.0914, 146.0651
133	Sanjoinine F	$C_{31}H_{42}N_4O_5$	$[M + HCOOH-H]^-$	595.31263	595.31244	−0.3	20.04	-
134	Cocculolidine	$C_{15}H_{17}NO_3$	$[M + H]^+$	260.12812	260.12852	1.6	20.67	83.0128, 162.0554, 176.0710, 188.0336, 204.0659, 260.1292
135	Atanine	$C_{15}H_{17}NO_2$	$[M + H]^+$	244.13321	244.13359	1.6	22.62	188.0708, 158.0599, 144.0472, 172.0399
**Saponins**								
136	Sanchinoside B1	$C_{36}H_{62}O_9$	$[M + H]^+$	639.44666	639.44620	−0.7	10.80	405.3014, 109.1011, 127.1119, 621.4437, 107.0845, 603.4266
137	Yesanchinoside D	$C_{44}H_{74}O_{15}$	$[M + HCOOH-H]^-$	887.49988	887.50395	4.6	15.34	781.4798, 637.4346, 619.4241, 799.4910, 101.0230, 161.0454, 113.0239, 475.3817, 119.0353, 783.4859, 801.4947
138	Yesanchinoside F	$C_{56}H_{94}O_{24}$	$[M − H]^-$	1149.60623	1149.61086	4.0	20.06	1107.6017, 1089.5908, 945.5491, 783.4942 , 621.4404
139	Araloside A	$C_{47}H_{74}O_{18}$	$[M − H]^-$	925.48024	925.48309	3.1	20.55	-
140	Ciwujianoside D1	$C_{55}H_{88}O_{22}$	$[M + H]^+$	1101.58400	1101.58154	−2.2	21.48	789.4762, 335.0947, 365.1035, 203.0540, 921.5035
**2,2-Dimethylchromenes**								
141	Leptol B	$C_{15}H_{20}O_4$	$[M + H]^+$	265.14344	265.14390	1.8	6.00	83.0461, 165.0690, 181.0657, 189.1289, 195.0799, 199.0764, 203.1043
142	Isoevodionol	$C_{14}H_{16}O_4$	$[M − H]^-$	247.09758	247.09782	0.9	8.88	173.0586, 135.0436, 187.077552, 107.0483, 133.0635, 151.0768, 135.0535, 189.0499
143	Leptonol	$C_{15}H_{18}O_5$	$[M − H]^-$	277.10815	277.10843	1.0	16.36	231.1026, 97.0292, 205.1212
**Others**								
144	Mannose	$C_6H_{12}O_6$	$[M − H]^-$	179.05611	179.05634	1.3	1.17	89.0238, 101.0232, 89.0323, 131.0350
145	Xylose	$C_5H_{10}O_5$	$[M − H]^-$	149.04555	149.04578	1.6	1.21	-
146	Sucrose	$C_{12}H_{22}O_{11}$	$[M + HCOOH-H]^-$	387.11332	387.11428	2.5	1.21	89.0254, 119.0355, 101.0252, 179.0567, 161.0459, 143.0354, 131.0353, 149.0457, 221.0673, 281.0871

Table S1. *Cont.*

No.	Compound	Formula	Ion Adduction	Theoretical *m*/*z* (Da)	Measured *m*/*z* (Da)	Error/ ppm	t_R/min	Product Ions (*m*/*z*, Da)
147	Dianthoside	$C_{12}H_{16}O_8$	$[M + H]^+$	289.09179	289.09178	−0.1	1.22	-
148	Manninotriose	$C_{18}H_{32}O_{16}$	$[M + H]^+$	505.17631	505.17591	−0.8	1.23	-
149	Caffeine	$C_8H_{10}N_4O_2$	$[M + HCOOH-H]^-$	239.07748	239.07732	−0.7	1.33	-
150	Safrole	$C_{10}H_{10}O_2$	$[M + H]^+$	163.07536	163.07532	−0.2	1.73	117.0698, 145.0670
151	Prodelphinidin B/ isomer	$C_{30}H_{26}O_{14}$	$[M − H]^-$	609.12498	609.12592	1.5	2.55	-
152	4-Hydroxycoumarin	$C_9H_6O_3$	$[M + H]^+$	163.03897	163.03903	0.4	5.10	-
153	Catechin	$C_{15}H_{14}O_6$	$[M + H]^+$	291.08631	291.08633	0.1	5.21	123.0444, 139.0396, 93.0335, 111.0447, 83.0497, 121.0288, 165.0549, 207.0653
			$[M − H]^-$	289.07176	289.07192	0.5	5.21	123.0459, 109.0304, 151.0404, 137.0252, 97.0310, 125.0249, 187.0409, 205.0505, 149.0244, 135.0463
154	Normelicopidine	$C_{16}H_{13}NO_5$	$[M + H]^+$	300.08665	300.08672	0.2	5.58	103.0523, 106.0292, 107.0505, 222.0598, 226.0866, 254.0808, 256.0592, 284.0544, 300.0858
155	Zizybeoside I	$C_{19}H_{28}O_{11}$	$[M − H]^-$	431.15589	431.15618	0.7	6.64	101.1233, 161.0441, 89.0237
156	Lyoniresinol	$C_{22}H_{28}O_8$	$[M + H]^+$	421.18569	421.18589	0.5	6.82	-
			$[M − H]^-$	419.17114	419.17114	0.0	6.82	357.1705, 199.1100, 263.1291, 237.1280, 255.1414, 135.0441, 317.1385
157	Blumenol A	$C_{13}H_{20}O_3$	$[M + H]^+$	225.14852	225.14838	−0.6	8.00	81.0687, 85.0279
158	Corchoionoside B	$C_{19}H_{28}O_9$	$[M − H]^-$	399.16606	399.16581	−0.6	9.17	219.1009
159	Geranyl acetate	$C_{12}H_{20}O_2$	$[M − H]^-$	195.13905	195.13902	−0.2	9.67	-
160	Evodone	$C_{10}H_{12}O_2$	$[M + H]^+$	165.09101	165.09109	0.5	12.63	81.0332, 109.0924
161	Physalin B	$C_{28}H_{30}O_9$	$[M + H]^+$	511.19626	511.19500	−2.5	13.21	-
162	Icariside B1	$C_{19}H_{30}O_8$	$[M + HCOOH-H]^-$	431.19117	431.19320	4.7	13.33	265.0937, 103.0405, 137.0616, 247.0832, 207.1039, 181.1243, 165.0916, 145.0513, 89.0256, 119.0350, 125.0606
163	Zizyvoside I	$C_{25}H_{40}O_{12}$	$[M − H]^-$	531.24470	531.24452	−0.3	15.68	-
164	α-Curcumene	$C_{15}H_{22}$	$[M + H]^+$	203.17943	203.17953	0.5	15.13	91.0540, 119.0851, 105.0692, 117.0697, 133.1014, 145.1013,
165	Panaxene	$C_{15}H_{24}$	$[M + H]^+$	205.19508	205.19522	0.7	19.82	-
166	Taraxerone	$C_{30}H_{48}O$	$[M + H]^+$	425.37779	425.37741	−0.9	20.37	123.1165, 189.1634, 203.1788, 217.1936, 207.1736, 191.1792, 271.2420, 137.1335, 285.2578, 151.1465, 205.1954
167	Panaxydol	$C_{17}H_{24}O_2$	$[M + H]^+$	261.18491	261.18511	0.8	21.02	91.0546, 103.0547, 117.0701, 131.0489, 137.0601, 145.0656, 147.0471, 151.0778, 161.0615, 173.0958, 175.0801
168	Copaene/isomer	$C_{15}H_{24}$	$[M + H]^+$	205.19508	205.19528	1.0	21.23	-
